# Prenatal Clinical Findings in *RASA1*-Related Capillary Malformation-Arteriovenous Malformation Syndrome

**DOI:** 10.3390/genes14030549

**Published:** 2023-02-22

**Authors:** Emanuele Coccia, Lara Valeri, Roberta Zuntini, Stefano Giuseppe Caraffi, Francesca Peluso, Luca Pagliai, Antonietta Vezzani, Zaira Pietrangiolillo, Francesco Leo, Nives Melli, Valentina Fiorini, Andrea Greco, Francesca Romana Lepri, Elisa Pisaneschi, Annabella Marozza, Diana Carli, Alessandro Mussa, Francesca Clementina Radio, Beatrice Conti, Maria Iascone, Giancarlo Gargano, Antonio Novelli, Marco Tartaglia, Orsetta Zuffardi, Maria Francesca Bedeschi, Livia Garavelli

**Affiliations:** 1 Medical Genetics Unit, Azienda USL-IRCCS di Reggio Emilia, 42123 Reggio Emilia, Italy; 2 Department of Medical and Surgical Science, Postgraduate School of Medical Genetics, Alma Mater StudiorumUniversity of Bologna, 40126 Bologna, Italy; 3 Paediatrics Unit, Azienda USL-IRCCS di Reggio Emilia, 42123 Reggio Emilia, Italy; 4 Neonatal Intensive Care Unit, Azienda USL-IRCCS di Reggio Emilia, 42123 Reggio Emilia, Italy; 5 Postgraduate School of Paediatrics, University of Modena and Reggio Emilia, 41121 Modena, Italy; 6 Translational Cytogenomics Research Unit, Laboratory of Medical Genetics, Bambino Gesù Children’s Hospital, IRCCS, 00146 Rome, Italy; 7 Medical Genetics Unit, Careggi University Hospital, 50134 Florence, Italy; 8 Medical Genetics Unit, Department of Experimental and Clinical Biomedical Sciences “Mario Serio”, University of Florence, 50121 Florence, Italy; 9 Department of Public Health and Pediatric Sciences, Regina Margherita Children’s Hospital, Azienda Ospedaliero-Universitaria di Torino, 10126 Turin, Italy; 10 Molecular Genetics and Functional Genomics, Bambino Gesù Children’s Hospital, IRCCS, 00146 Rome, Italy; 11 Clinical Genetics Unit, Fondazione IRCCS Ca’ Granda Ospedale Maggiore Policlinico, 20122 Milan, Italy; 12 Laboratory of Medical Genetics, Ospedale Papa Giovanni XXIII, 24127 Bergamo, Italy; 13 Unit of Medical Genetics, Department of Molecular Medicine, University of Pavia, 27100 Pavia, Italy

**Keywords:** capillary malformation-arteriovenous malformation (CM-AVM), *RASA1*, prenatal findings, polyhydramnios, non-immune fetal hydrops, chylothorax

## Abstract

Pathogenic variants in *RASA1* are typically associated with a clinical condition called “capillary malformation-arteriovenous malformation” (CM-AVM) syndrome, an autosomal dominant genetic disease characterized by a broad phenotypic variability, even within families. In CM-AVM syndrome, multifocal capillary and arteriovenous malformations are mainly localized in the central nervous system, spine and skin. Although CM-AVM syndrome has been widely described in the literature, only 21 cases with prenatal onset of clinical features have been reported thus far. Here, we report four pediatric cases of molecularly confirmed CM-AVM syndrome which manifested during the prenatal period. Polyhydramnios, non-immune hydrops fetalis and chylothorax are only a few possible aspects of this condition, but a correct interpretation of these prenatal signs is essential due to the possible fatal consequences of unrecognized encephalic and thoracoabdominal deep vascular malformations in newborns and in family members carrying the same *RASA1* variant.

## 1. Introduction

Capillary malformation-arteriovenous malformation type 1 (CM-AVM 1, MIM 608354) is a genetic autosomal dominant disorder first described in 2003 by Eerola et al., who identified heterozygous inactivating variants of the *RASA1* gene in six out of seventeen families in which individuals presenting with capillary malformations were reported [1].

*RASA1* encodes the p120 RasGAP protein, a GTPase activating protein functioning as negative regulator of the RAS GTPases and the downstream mitogen-activated protein kinase (MAPK) signal transduction pathway [2]. *RASA1* haploinsufficiency leads to increased cellular proliferation and survival, mainly in endothelial cells of the vascular system [3,4,5]. As a result, multifocal capillary malformations (CMs), arteriovenous malformations (AVMs) and arteriovenous fistulas (AVFs) can occur, affecting the skin and the central nervous system or thoracoabdominal deep blood vessels, with risk of complications when fast-flow lesions occur. The condition is characterized by great phenotypic inter- and intra-familial variability, and it may occur either with small cutaneous “port-wine stains” or with more severe clinical features, such as high-flow AVMs and/or lymphatic anomalies. Some individuals with CM-AVM syndrome may exhibit phenotypic features compatible with Parkes Weber syndrome, which is characterized by the presence of multiple micro-AVFs associated with underlying soft tissue and skeletal overgrowth. When the clinical features are severe, complications can potentially be fatal, as in the case of acute hemorrhagic cardiovascular events or congestive heart failure [6,7,8].

To elucidate the molecular mechanisms underlying the vascular and lymphatic abnormalities resulting from *RASA1* haploinsufficiency, in 2014, Lubeck and colleagues generated a mouse model for Rasa1 loss of function (R780Q) by removal of the arginine residue required for the RAS-specific GTPase activity [2]. This amino acid substitution resulted in a protein that was specifically unable to exert its negative regulation on RAS GTPases, without perturbing the RAS-independent functions of the GAP [3,4]. The lethal phenotype caused by severe vascular anomalies occurring during gestation was similar to those observed in *RASA1*-null mice [9], suggesting that CM-AVM syndrome is likely caused by the loss of ability of the GAP to properly regulate the RAS/MAPK pathway [3].

From a molecular point of view, CM-AVM syndrome shares the upregulation of the RAS-MAPK signaling cascade with RASopathies; on the other hand, it is not characterized by multisystemic involvement. Though a general consensus is lacking, recent publications placed this disorder in the group of the “nonsystemic RASopathies” [10,11,12,13].

While more than 300 individuals affected by the condition have been reported worldwide [14], prenatal manifestations of CM-AVM syndrome have been rarely discussed. The condition is probably underdiagnosed, since our knowledge of its antenatal and neonatal presentation is still poor, with only 21 cases with prenatal onset of the disorder described to date [11,14,15,16,17,18,19,20,21,22,23,24,25,26,27].

Here, we describe four previously unreported pediatric cases showing a prenatal onset of the disorder, and a literature review of previously reported cases. This report contributes to strengthen and expand the knowledge of the clinical spectrum of *RASA1* [28], pointing out the importance of considering the prenatal clinical signs to guide the diagnosis of this condition.

## 2. Materials and Methods

The probands’ parents provided written informed consent for molecular analyses and publication of the results and photographs.

The analyses were focused on disease-associated genes based on the proband’s clinical features, and considered either X-linked, autosomal recessive or autosomal dominant transmission patterns.

Patient 1: Clinical exome sequencing (CES) was performed on genomic DNA extracted from circulating leukocytes of the proband and their parents using standard procedures. Library preparation was carried out by using the Twist Custom Panel kit (Twist Bioscience, South San Francisco, CA, USA) according to the manufacturer’s protocol and sequenced on a NovaSeq6000 (Illumina, San Diego, CA, USA) platform. The target parameters included the coding exons with a region extension of 25 bases from the 3′ end and 25 bases from the 5′ end (based on RefSeq database). The target regions had a mean 150× coverage, a specificity of 100% and a sensitivity of 100%, with a quality score of ≥30. A minimum depth coverage of 30× was considered suitable for analysis. The BaseSpace pipeline and the TGex software LifeMap Sciences v.3 were used for variant calling and annotation, respectively. Sequencing data were aligned to the hg19 human reference genome. Analysis was performed in the trio using a custom “virtual” panel comprehensive of all genes currently associated with RASopathies or capillary malformations.

Confirmation and familial segregation of the identified pathogenic *RASA1* variant were performed by Sanger sequencing (FastStartTaq DNA Polymerase, Sigma-Aldrich, St. Louis, MO, USA). Primer sequences are listed in Supplementary Materials Table S1. The PCR products were sequenced using a Big Dye Terminator v1.1 Cycle Sequencing Kit (Thermo Fisher Scientific, Waltham, MA, USA) and run on an ABI3500 Dx Genetic Analyzer (Thermo Fisher Scientific).

Total RNA was isolated from peripheral blood lymphocytes of family 1 using an RNeasy Mini Kit (Qiagen, Hilden, Germany). A total of 1 μg of RNA was reverse transcribed into cDNA using random hexamer and oligodT primers and a Transcriptor First Strand cDNA Synthesis kit (Roche Diagnostics, Rotkreuz, Switzerland). Evaluation of the variant’s effect on the mRNA was assessed by Sanger sequencing. Quantitative real-time was performed using a CFX96 Touch Real-Time PCR Detection System (Bio- Rad Laboratories, Hercules, CA, USA) and TB Green Premix Ex Taq II Mix (Takara Bio, Kusatsu, Shiga, Japan) according to the manufacturer’s instructions. The primer sequences are listed in Supplementary Materials Table S1. For quantification of genes expression, ACTB was used as an endogenous control. Relative quantification was performed using the comparative method. The results were expressed using the DDCt method.

Patient 2: Whole exome sequencing (WES) was performed on genomic DNA extracted from circulating leukocytes of the proband and their parents using standard procedures. Regions containing exons and flanking intronic regions were enriched using a SureSelect Clinical Research Exome kit (Agilent) and analyzed by parallel sequencing (Illumina, PE 2 × 150). A targeted NGS assay that has a mean 194× and >10× coverage in 99.5% of the target regions was obtained. Identified variations were classified according to the ACMG guidelines and that included in the report was confirmed by an alternative method and reported according to the HGVS nomenclature. The bioinformatic analyses were performed using BWA version 0.7.17-r1188 (released 23 October 2017), SAMtools version 1.9 (released 18 July 2018), Picard tools version 2.21.2 (released 28 October 2019) and GATK version 3.8-1 (released September 2017). Sequencing data were aligned to the hg19 human reference genome. 

Patient 3: CES was performed as described for Patient 1. A mean 351.99× coverage, with a specificity and sensitivity of >99% and a quality score ≥ 30, was obtained. A minimum depth coverage of 30× was considered suitable for analysis. Sequencing data were aligned to the hg19 human reference genome, and the Geneyx Anaylisis Software (Knowledge-Driven NGS Analysis tool powered by the GeneCards Suite) was used for the filtering and prioritization of variants.

Patient 4: The study of the coding regions and exon-intron junctions of the *RASA1* gene was performed on genomic DNA extracted from circulating leukocytes of the proband and their parents using standard procedures. The target regions were enriched using the NimbleGen SeqCap Target Enrichment kit (Roche) and then sequenced on NovaSeq 6000 platform (Illumina). Confirmatory testing was performed by Sanger amplification and sequencing with automated capillary sequencer. Analysis of the generated data was performed using Isis (Analysis Software) 2.5.1.3, BWA (Aligner) 0.6.1-r104-tpx, SAMtools 0.1.18 (r982:295) and GATK (Variant Caller) systems, implemented in the Illumina protocol. Sequencing data were aligned to the hg19 human reference genome.

## 3. Patients and Results

### 3.1. Patient 1

The proband is a girl, the third child of healthy non-consanguineous parents, both of south Asian origin. The pregnancy was complicated by gestational diabetes. Obstetric ultrasound (US) at the 28th week showed bilateral pleural and severe pericardial effusion with non-immune hydrops fetalis and polyhydramnios, requiring a thoracic-amniotic shunt. CGH-array analysis performed on amniocytes showed a normal 46, XX karyotype.

The proband was born preterm at 33 weeks and 4 days of gestation from a cesarean section with a birth weight of 1920 g (41st centile), length of 45 cm (71st centile) and occipital-frontal circumference (OFC) of 27.5 cm (2nd centile). The Apgar scores were 4 and 8, respectively, at 1 and 5 min after birth. Cardio-pulmonary resuscitation was required with rapid recovery.

**Figure 1:**
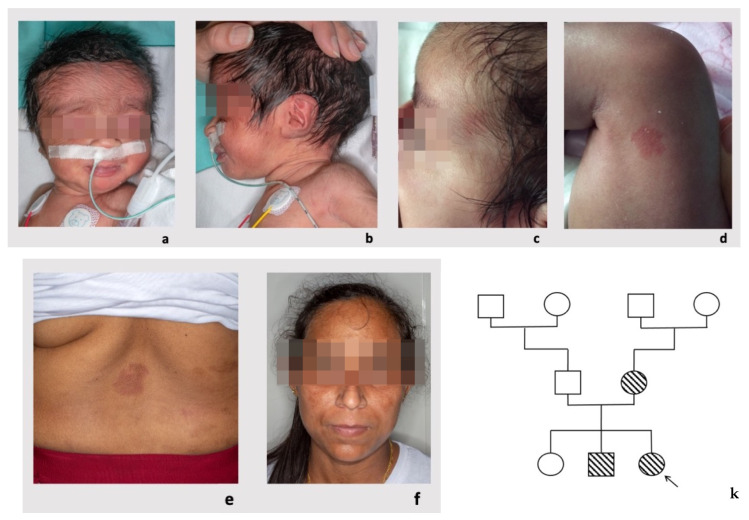

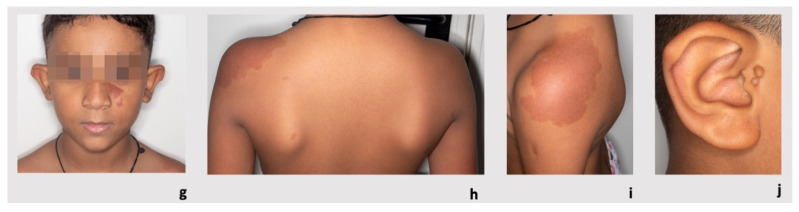
Patient 1. Proband: clinical features at 80 days: small capillary malformations of round or oval shape were evident on the face (**a**–**c**) and lower limbs (**d**). Mother: Capillary malformations on the face and thoracolumbar region (**e**,**f**). Brother: Capillary malformations on the face and left shoulder (**g**–**i**); pre-auricular tags on the tragus involving the right ear (**j**). Pedigree of Patient 1’s family (**k**).

Clinical evaluation at birth showed mild hypertrichosis of the forehead and posteriorly rotated ears with an overfolded helix on the left side. A microscopic analysis of the pleural fluid on a sampling performed at 6 days of life was carried out, finding a severe increase in nucleated cell numbers and the triglycerides value, features consistent with a diagnosis of chylothorax. The rounded/oval small capillary malformations, which were not evident at birth, were first noticed at 2 months and 20 days of life (Figure 1a–d). The postnatal clinical history was complicated by the persistence of pleural effusion and hydropericardium; thoracoscopy-thoracotomy was necessary, with cleaning of the pleural cavity and detection of a red-purple 2–3 cm angiomatous growth at the level of the posterior-superior wall of the pleural cavity. Echocardiography showed patent ductus arteriosus (PDA). Intracranial arterial MRI angiography was normal.

CES analysis identified a single nucleotide deletion at the heterozygous state, NM_002890:c.2923del, causing premature termination of the coding sequence in the second-last exon (24/25) of *RASA1*, p.(Asn976fs*19). The variant, inherited from the mother, was not reported in public databases (gnomAD v2.1.1, https://gnomad.broadinstitute.org/, accessed on 12 December 2022) and was classified as pathogenic according to the guidelines of the College of American Genetics and Genomics/Association for Molecular Pathology (ACMG/AMP) (Richards et al., 2015) [29,30,31,32].

Segregation analysis identified the same heterozygous *RASA1* variant in the proband’s mother and brother.

The proband’s mother was healthy and did not report any significant hospitalization or surgeries. Her first two pregnancies had physiological evolution, without prenatal complications, and resulted in birth at term of a daughter and a son. On physical examination, she presented some capillary vascular anomalies (Figure 1e,f) that appeared as rounded or oval erythematous macules on the back (diameter of 4 cm and 2 cm, respectively) and on the right shoulder (diameter of 3 cm).

The proband’s brother was also healthy and did not report any significant hospitalizations or surgeries. He was born at term from a vaginal delivery after a physiological pregnancy. Prenatal US scans were normal. On physical examination, at 8 years of age, he presented with posteriorly rotated ears and pre-auricular tags on the tragus involving the right ear. He showed capillary vascular anomalies (Figure 1g–j) that appeared as rounded/oval erythematous macules on the left side of the nasal root (diameter 2.8 cm × 0.8 cm × 0.8 cm), on the left shoulder (diameter 13 cm × 8.5 cm), on the neck and on the lower limbs (five spots with diameter < 1 cm). He had a normal psychomotor development and growth parameters.

**Figure 2:**
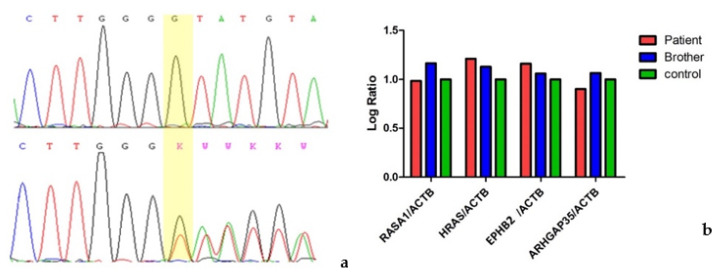
Electropherogram–affected brother below, unaffected sister above (**a**); Performing Real Time PCR, we evaluated the expression levels of *RASA1* and of other genes directly regulated in pathways in which *RASA1* is involved (*HRAS*, *EPHB2*, *ARHGAP35*), that showed an overlapping gene expression (including RASA1 gene) in proband, affected sibling and three independent controls (**b**).

We performed an RNA analysis which showed biallelic expression (Figure 2a), confirming that PTC did not undergo degradation of the transcript by nonsense-mediated decay (NMD).

We then investigated whether the pathogenic variant could lead to a reduction in *RASA1* mRNA expression and furthermore we selected three pathways in which *RASA1* is involved: RAS/MAP kinase cascade, Ephrin signaling and *PTK6* signaling. For each pathway we evaluated the expression of the genes directly related to *RASA1* gene: *HRAS* (NM_005343.4), *EPHB2* (NM_017449.5) and *ARHGAP35* (NM_004491.5), respectively. No statistically significant differences were identified between variant carriers (proband and her brother) and controls (Figure 2b).

### 3.2. Patient 2

Patient 2 is a boy, firstborn of healthy, non-consanguineous parents, both of Caucasian origin. Family history was initially uneventful.

The US at 19 weeks of gestation detected polyhydramnios, requiring amniodecompression, and increased fetal biometry (>95th centile) in the absence of major congenital malformations (MCA). Karyotype and array-CGH on amniocytes revealed a normal 46, XY karyotype without significant structural variants. Gestational diabetes was also excluded.

He was born at 35 weeks and 1 day of gestation by spontaneous delivery. His parameters at birth were weight: 3780 g (>97th centile), length: 49.5 cm (>90th centile) and OFC: 36.5 cm (>97th centile). The Apgar scores were 8 and 9, respectively, at 1 and 5 min. The baby was admitted to the NICU for hypotonia and breathing difficulties.

MRI, performed at birth and repeated at 2 years of age, showed small posterior fossa with cerebellar tonsils descending through the foramen magnum, with features consistent with a Chiari type I malformation, associated with dysmorphism of the corpus callosum and lateral ventricular cavities, immaturity of the white matter and a suspicion of altered cortical development (polymicrogyria).

A clinical follow up at 3 years of age indicated that the facial features had become coarser and noted an eruptive angiomatosis compatible with CM-AVM syndrome, as well as five café-au-lait spots on the upper and lower limbs. The overgrowth persisted in the postnatal period with macrocephaly and a rapid increase in the OFC (55 cm at two years of age, >97th centile). There was no evidence of intellectual disability. An ophthalmological examination, cardiac and abdominal USs and spine MRI were normal.

A targeted NGS panel for overgrowth syndromes, including *CDKN1C*, *DIS3L2*, *GPC3*, *NSD1*, *NFIX*, *OFD1*, *EZH2*, *PTEN*, *IGF2* and MLPA of the *PTEN* gene, did not identify any pathogenic variant.

Upon suspicion of an RASopathy during the clinical follow up, trio WES analysis was performed, revealing the previously unreported heterozygous variant NM_002890.3:c.693-5A>G in the *RASA1* gene. This variant, which was maternally inherited, involves intron 1 and was predicted to alter splicing [33], is absent in gnomAD v2.1.1, and could be classified as likely pathogenic according to the ACMG/AMP guidelines (Richards et al., 2015) [29,30,31,32].

Reverse phenotyping in the mother revealed the presence of several cutaneous anomalies compatible with CM/AVM syndrome.

### 3.3. Patient 3

Patient 3 is a girl, the second child of non-consanguineous parents. During pregnancy, combined screening in the first trimester indicated a high risk for trisomy 21 and trisomy 13, with the US revealing an increased nuchal translucency (3.4 mm) and a single umbilical artery. However, the QF-PCR, karyotype and CGH-array performed on a chorionic villus sample were normal. The subsequent US exams showed a flat fetal profile with normal nasal bone size at the 20th week and polyhydramnios at the 22nd week. CES performed on fetal and parental DNA detected the heterozygous variant NM_002890.3:c.1052G>A in the *RASA1* gene, which at the protein level determines the introduction of the premature stop codon, p.(Trp351*). This nonsense variant, inherited from the mother and not described in the scientific literature before, is absent in gnomAD v2.1.1, and can be classified as pathogenic according to the ACMG/AMP guidelines [29,30,31,32].

The patient was born at 38 weeks of gestation with a birth weight of 3370 g (70th centile), a length of 47 cm (14th centile), an OFC of 34 cm (55th centile) and Apgar scores of 9 and 10 at 1 and 5 min, respectively.

A clinical evaluation revealed the presence of two capillary malformations in the abdominal and lumbar regions, and further dermatological examinations performed at three weeks of age found four capillary malformations in the retronuchal region, dorsum, right thigh and left thigh root. In addition, assessment by a genetic counselor at one month of age detected peculiar facial features, consisting of upslanting palpebral fissures, a broad root and nasal bridge, hypoplastic and anteverted nostrils, uplifted earlobes and overfolded helices. Intracranial arterial MRI angiography showed a prominence of the vascular structures at the temporo-polar region in the absence of arteriovenous shunts, while echocardiography revealed a physiologic patent foramen ovale (PFO). An abdominal US, Auditory Brainstem Response testing and ophthalmologic evaluations were normal.

A clinical examination of the mother confirmed the presence of superficial capillary malformations compatible with CM-AVM syndrome.

### 3.4. Patient 4

Patient 4 is a boy, the first child of non-consanguineous parents, both of eastern European origin. Pregnancy was followed in the country of origin until the 31st week of gestation, with prenatal evidence of cardiomegaly and high-flow arteriovenous malformation of the right upper limb associated with an increased thickness of muscle and skin tissues and an increased length of the humerus and ulna. The first obstetric ultrasound (performed in Italy at 32 weeks and 5 days of gestation) showed bilateral moderate–severe pleural effusion, mild ascitic effusion, cardiomegaly with moderate tricuspid valve insufficiency and reverse-flow at the level of the aortic arch.

The patient was born at 33 weeks of gestation with a birth weight of 2490 g (98th centile), a length of 44 cm (74th centile), an OFC of 34 cm (>97th centile, +2.9 SDS) and Apgar scores of 2 and 5 at 1 and 10 min, respectively.

**Figure 3:**
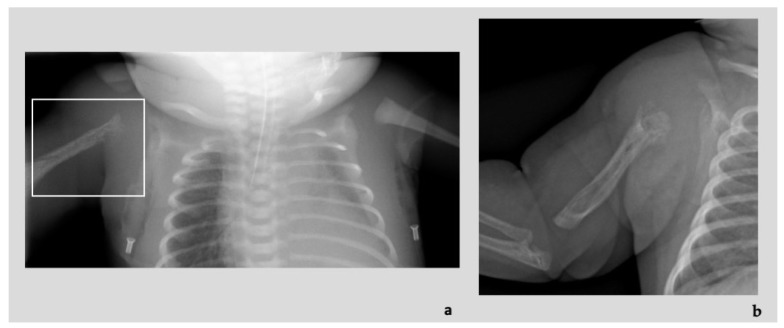

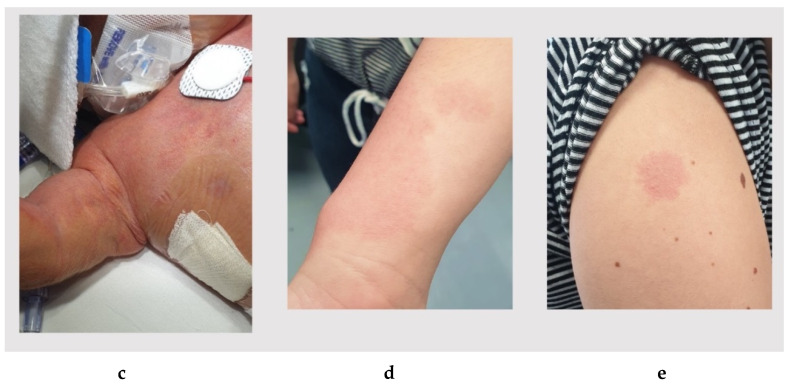
Patient 4: Radiographic images showing destruction and absorption of the proximal epiphysis of the humerus (adjacent to the arteriovenous malformation of the right arm), features indicative of Parkes Weber Syndrome ((**a**) at birth and (**b**) at 2 months of life). Proband at birth with overgrown and swollen right limb with skin port-wine stains and subcutaneous pulsatile vascular anomalies (**c**); proband’s mother capillary malformations on her wrist and shoulder (**d**,**e**).

Diagnostic imaging investigations showed the presence of numerous ectatic and tortuous high-flow arterial structures at the level of the vascular malformation of the right upper limb, associated with diffuse osteostructural alteration of the humerus and proximal radius and ulna (Figure 3a,b). At the clinical evaluation at birth, the newborn presented edematous with respiratory distress, heart failure consequent to blood shunt, an overgrown and swollen right limb with skin port-wine stains and subcutaneous pulsatile vascular anomalies (Figure 3c). An echocardiogram also showed dilatation of the superior vena cava and of the right cardiac sections, patent foramen ovale (PFO) with right-left shunt. He was admitted to the neonatal intensive care unit where he was intubated and treated with prostaglandins, steroid and beta-blocker drug therapy with progressive improvement in the clinical features. The vascular malformations were treated with endovascular coil insertion.

The clinical features raised the suspicion of CM-AVM syndrome presenting with a Parkes Weber syndrome-like phenotype, which was molecularly confirmed by the presence of the heterozygous variant NM_002890.2:c.768C>A in the *RASA1* gene. This nonsense variant, which determines the introduction of a premature stop codon in exon 3 and would translate as p.(Tyr256*) at the protein level, is absent in gnomAD v2.1.1 (accession 12/2022) and can be classified as pathogenic according to the ACMG/AMP guidelines [29,30,31,32].

A segregation analysis revealed the presence of the same heterozygous *RASA1* variant in the mother, which showed only three flat pink capillary malformations on the skin of wrist, shoulder and ankle (Figure 3d,e).

### 3.5. Review of the Literature

To date, a prenatal presentation of CM-AVM syndrome has been described only in 21 individuals with pathogenic *RASA1* variants. The most frequent prenatal presentations are summarized in Table 1.

Prenatal signs are often diagnosed by ultrasonography; according to the literature, polyhydramnios was detected in 38.1% of prenatal cases of CM-AVM syndrome Table S2, pleural effusion and non-immune hydrops fetalis in 23.8%, ascites in 10% and pericardial effusion in 5%. In the present cohort, the most frequent anomaly in the prenatal period was polyhydramnios (three out of four cases). Among the consequences related to the presence of lymphatic vessel abnormalities, chylothorax is the most frequently reported in patients with prenatal onset of symptoms (15%).

As expected, the main clinical features seem to be better recognized postnatally. Among these, skin capillary malformations represent the most frequent feature, present in 11 out of 15 cases (73.3%), recapitulated in our patients. Vascular malformations were described in 13 out of 20 (65%) patients, as in our patients 1 and 4. Our patient 4, in particular, presented with osteostructural and soft tissue anomalies associated with multiple AVMs, configuring Parkes Weber Syndrome, reported in 12.5% of the analyzed cohort. Heart failure occurred in 25% of reported patients, while structural cardiac anomalies were present in 4 out 21 (19%) patients.

In four cases, including our patients 1 and 4, CM-AVM syndrome presented in such a severe prenatal form with polyhydramnios and hydrops fetalis as to require in utero drainage/shunt, with favorable evolution in our patients and a second case [23] and death in another case [24]. In patients in whom the condition arose prenatally, death occurred in six cases (30%). However, this may be an expression of the great phenotypic variability of CM-AVM syndrome. Furthermore, because of this variability, we found no significant correlation between prenatal manifestations and postnatal severity.

**Figure 4:**
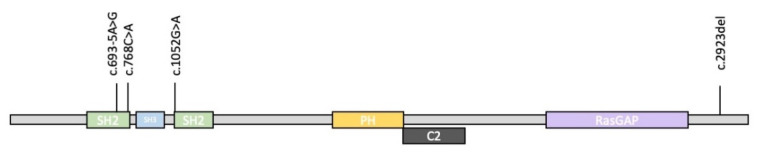
Schematic representation of the localization of variants found in our patients in the *RASA1* gene: one splicing variant (c.693-5A>G), two nonsense variants (c.768C>A and c.1052G>A) and one frameshift variant (c.2923del).

Information about mutations of the *RASA1* gene are listed in Table 2, in comparison with our patients’ mutation features. Our data confirm those reported in the literature, whereby the condition is mainly caused by nonsense and frameshift variants or, to a lesser extent, by the presence of variants altering the splicing. Truncating variants can affect all regions of the gene (Figure 4). All our patients inherited the *RASA1* pathogenic variant from their mother.

## 4. Discussion

Prenatal suspicion of RASopathies may arise from peculiar US features, including increased nuchal translucency, cystic hygroma, fetal hydrops, pleural effusion, ascites, a distended jugular lymphatic sac, cardiac anomalies, renal abnormalities and polyhydramnios [11,34,35]. These features, isolated or combined, are considered as indications for genetic testing [11,34,36]. In particular, the diagnosis of an underlying RASopathy appears to be significantly associated with the severity and timing of polyhydramnios [11]. Therefore, early identification of the possible prenatal manifestations of these conditions allows not only to induce diagnostic suspicion, but also to improve the prevention of critical perinatal issues, including respiratory and cardiac complications.

In this regard, the data from our patients and from the literature suggest that although CM-AVM syndrome does not entirely fit the clinical spectrum of systemic RASopathies, it can present with some overlapping signs, even in the prenatal period.

The rarity of reports on prenatal symptoms of CM-AVM syndrome indicates that they are either unusual or, more likely, rarely recognized and therefore under-reported. By describing the prenatal characteristics of our four cases and providing a literature review, our aim was to identify the possible elements that can guide the clinicians towards an early diagnosis of this condition and to focus on aspects of the disease that are often underestimated. Ultrasound is the most accurate tool for identifying eventual signs of the disease in the prenatal period. In particular, it is important to pay attention to polyhydramnios, non-immune hydrops fetalis (pleural effusion, pericardial effusion and ascites), congestive heart failure and detection of vascular malformations. AVMs are frequent clinical features which deserve particular attention since the consequences of an undiagnosed vascular malformation could be potentially fatal. Some of these vascular abnormalities, in fact, can only be diagnosed by performing a second level radiological examination (such as CT), as was the case for our Patient 1. Not surprisingly, our two patients with high-flow vascular malformations also presented with severe heart failure, which resulted in a long hospitalization in the neonatal intensive care unit; in particular, our Patient 4 presented with cardiomegaly as cardiac structural damage resulting from the AVM. The typical stains caused by CMs can be evident after birth or in the first months of life, as was the case, for example, in our Patient 1, in whom they appeared at the age of 2 months and 20 days. In any case, they represent the most frequently occurring clinical feature in patients affected by CM-AVM syndrome, even in the cohort of patients in whom the condition arose prenatally.

In the presence of prenatal US elements suggestive of an RASopathy, it is recommended to provide genetic counseling and to consider CM-AVM syndrome in the differential diagnosis with the systemic RASopathies. It may also be useful to accurately examine the family members to exclude the presence of skin CMs. The simultaneous presence of specific US anomalies in the proband and CMs in first-degree relatives should suggest including molecular analysis of *RASA1* in the genetic testing, either prenatally or postnatally.

Identifying the condition in the prenatal or perinatal period is essential to carry out the most appropriate early care and to perform radiological diagnostic investigations which can exclude the presence of vascular malformations in areas that generally are not otherwise examined, not only in newborns, but also in first-degree family members who may not know they are affected. Although this condition has generally a good long-term prognosis, it is necessary to consider the possible complications of undiagnosed high-flow vascular malformations (such as bleeding, heart failure and epilepsy). Our patients’ clinical history confirms the presence of a great phenotypic variability even within the same family; the mothers of all our patients and Patient 1’s brother, carriers of the same pathogenic *RASA1* variant detected in the probands, reported no prenatal anomalies and presented only with asymptomatic capillary malformations. However, it is necessary to provide second-level radiologic investigations also in these affected family members, to exclude the presence of undiagnosed high-flow vascular malformations. Further studies are needed to understand the mechanisms underlying such a variability of effects determined by the same variants in different family members, focusing on genotype-phenotype correlation and somatic second-hit mutations.

## Figures and Tables

**Table 1:** CM-AVM syndrome clinical features in previously reported cases [11,14,15,16,17,18,19,20,21,22,23,24,25,26,27], characterized by a prenatal onset of symptoms, in order of frequency and compared with our patients’ characteristics. Abbreviations: AVMs, arteriovenous malformations; AVF, arteriovenous fistulas; VGAM, vein of Galen aneurysmal malformations; PFO, patent foramen ovale; CM, cardiomegaly; n/r, not reported; P1-2-3-4, Patient 1-2-3-4.

Clinical Feature	Literature Review Frequency (%)	This Study P1	This Study P2	This Study P3	This Study P4	Total
	[11,14,15,16,17,18,19,20,21,22,23,24,25,26,27]					
Capillary malformations, postnatal findings (skin)	11/15 (73.3%)	+	+	+	+	15/19 (78.9%)
Increased fetal nuchal thickness	2/3 (66.7%)	-	-	+	n/r	3/6 (50%)
Vascular malformations, including postnatal findings (AVMs and AVFs)	13/20 (65%)	+	-	-	+	15/24 (62.5%)
Polyhydramnios	8/21 (38.1%)	+	+	+	-	11/25 (44%)
Deceased	6/20 (30%)	-	-	-	-	6/24 (25%)
Cardiac failure	5/20 (25%)	+	-	-	+	7/24 (29.2%)
Pleural effusion	5/21 (23.8%)	+	-	-	+	7/25 (28%)
Non-immune hydrops fetalis	5/21 (23.8%)	+	-	-	-	6/25 (24%)
Structural cardiac anomalies	4/21 (19%)	-	-	+ (PFO)	+ (CM, PFO)	6/25 (24%)
Chylothorax	3/20 (15%)	+	-	-	-	4/24 (16.7%)
Parkes Weber Syndrome	2/16 (12.5%)	-	-	-	+	3/20 (15%)
Ascites	2/20 (10%)	-	-	-	+	3/24 (12.5%)
In utero drainage/shunt	2/20 (10%)	+	+	-	-	4/24 (16.7%)
VGAM	2/20 (10%)	-	-	-	-	2/24 (8.3%)
Renal anomalies	2/21 (9.5%)	-	-	-	-	2/25 (8%)
Pericardial effusion	1/20 (5%)	+	-	-	-	2/24 (8.3%)
Basilar artery aneurism	1/20 (5%)	-	-	-	-	1/24 (4.2%)

**Table 2:** *RASA1* mutations featured in reported CM-AVM syndrome cases characterized by prenatal onset of symptoms [11,14,15,16,17,18,19,20,21,22,23,24,25,26,27] compared with our patients’ characteristics. Abbreviations: P1-2-3-4, Patient 1-2-3-4.

	Literature Review Frequency (%) [11,14,15,16,17,18,19,20,21,22,23,24,25,26,27]	This Study P1	This Study P2	This Study P3	This Study P4	Total
**Inheritance**						
De novo *RASA1* variant	4/17 (23.5%)	-	-	-	-	4/21 (19%)
Inherited *RASA1* variant	13/17 (76.5%)	+ (mother)	+ (mother)	+ (mother)	+ (mother)	17/21 (81%)
**Variant information**						
Nonsense variant	7/18 (39%)	-	-	+ c.1052G>A	+ c.768C>A	9/22 (41%)
Frameshift variant	7/18 (39%)	+ c.2923del	-	-	-	8/22 (36%)
Splicing variant	2/18 (11%)	-	+ c.693-5A>G	-	-	3/22 (14%)
Missense variant	1/18 (5.5%)	-	-	-	-	1/22 (4.5%)
Deletion	1/18 (5.5%)	-	-	-	-	1/22 (4.5%)

## Data Availability

The data that support the findings of this study are available from the corresponding author upon reasonable request.

## Supplementary Materials

The following supporting information can be downloaded at: https://www.mdpi.com/article/10.3390/genes14030549/s1, Table S1: List of primer sequences; Table S2: Additional clinical features and molecular data of reported patients affected by CM-AVM Syndrome with prenatal onset of symptoms. Abbreviations: pn, postnatal finding; CMs pn, capillary malformations (postnatal diagnosis); AVMs, arteriovenous malformations; AVF, arteriovenous fistulas; PWS pn, Parkes Weber Syndrome (postnatal diagnosis); VGAM, vein of Galen aneurysmal malformations; n/a, not applicable; n/r, not reported; PDA, patent ductus arteriosus; PFO, patent foramen ovale; AMI, Acute myocardial infarction; FCM, fetal cardiomyopathy; ^1^, unilateral pyelectasis; ^2^, pelviectasis/hydronephrosis; CM, cardiomegaly [11,14,15,16,17,18,19,20,21,22,23,24,25,26,27].
