# Supplementary material for: Prenatal Clinical Findings in RASA1-Related Capillary Malformation-Arteriovenous Malformation Syndrome
Source: Genes (Basel). 2023 Feb 22;14(3):549. doi: 10.3390/genes14030549 (PMC10048332; doi:10.3390/genes14030549)
Supplement: Supplementary file 1 [file genes-14-00549-s001.zip › Table S1_26.01.23.pdf]

Table S1. List of primer sequences.

**Name**	**Sequence 5’----3’**
RASA1_23F	TAATTACTTTTGCACCAACC
RASA1_23R	TTTGTTTAGGAAGGCAGAC
RASA1_cDNA_21-25F	CTTCGACTCATCTGTCCTG
RASA1_cDNA_21-25R	GAAGCAGTTCTGTTATAGCC
RASA1_RT_2-3F	ACGATAGCAGAAGAACGCC
RASA1_RT_2-3R	CCACCAATGTAGTAATCTCCAC
HRAS_RT_1-2F	GGAGCGATGACGGAATATAAG
HRAS_RT_1-2R	TGATGGCAAACACACACACAG
EPHB2_RT_7-8F	AAAAAGCCCCACCAACAC
EPHB2_RT_7-8R	TGACAACCACAGCAATGAG
ARHGAP35_RT_3-4F	AAACCAAAGCCCAAACCC
ARHGAP35_RT_3-4R	GCAGACTCTCCATCTCAGAC
ACTB_F	GCAAGAGATGGCCACGGCTG
ACTB_R	ATCCACACGGAGTACTTGCG
